# Colonic Atresia: Association with Other Anomalies

**DOI:** 10.21699/jns.v5i4.422

**Published:** 2016-10-10

**Authors:** Khaled M. El-Asmar, Mohammed Abdel-Latif, Abdel-Hamid A. El-Kassaby, Mohamed H. Soliman, Mosad M. El-Behery

**Affiliations:** 1 Pediatric Surgery Department, Ain Shams University, Cairo, Egypt.; 2 Pediatric Surgery Division, Helwan University, Egypt

**Keywords:** Colonic atresia, Intestinal atresia, Hirschsprung's disease, Associated anomalies

## Abstract

Background: Colonic atresia (CA) is a rare form of congenital intestinal atresia. Although CA may be isolated, it is more commonly reported in literature in association with other congenital anomalies.

Materials and Methods: This study is a review of prospectively collected data of all the patients with colonic atresia presented to our center (Ain Shams University) during 2008 to 2016.

Results: Twelve patients were enrolled in this study. The atresia was of type I in one case, type II in four cases, type IIIa in six cases, type IV in one case. These cases accounted for 4.9 % of intestinal atresias managed in our center during the same period. Five cases were isolated CA, while the other seven cases had associated abdominal congenital anomalies (exomphalos, Hirschsprung's disease, imperforate anus, closing gastroschisis, colonic duplication, and multiple small bowel atresia in two cases). The management in ten cases was by staged procedure with creation of a temporary stoma initially, while primary anastomosis was established in two cases. We had two cases with delayed presentations, one missed diagnosis, and three mortalities in this series.

Conclusions: The low incidence of CA may result in delay in the diagnosis and management. Hirschsprung's disease should be excluded in every case of colonic atresia. Early diagnosis and proper surgical management is essential for good prognosis.

## INTRODUCTION

Colonic atresia (CA) is a rare cause of congenital intestinal obstruction with a reported incidence of approximately 1:66,000 live births [1]. Since it is a rare condition, CA represents a challenge for the pediatric surgeons regarding its diagnosis and management. There is debate in the literature about the pathological theories for development of CA. One of the remarkable features of the CA is that it has a high incidence of associated congenital anomalies estimated to be 47% in the largest literature review [2]. Associated anomalies vary between abdominal anomalies (abdominal wall defect, multiple intestinal atresia, Hirschsprung's disease, and malrotation) and extra abdominal anomalies (musculoskeletal, ocular, and facial anomalies) [2]. Among these anomalies, the presence of associated Hirschsprung's disease represents a main issue in surgical decision making whether to do a primary anastomosis or to fashion a temporary stoma. Unawareness of that association while establishing a primary anastomosis usually leads to anastomotic dehiscence and complicated postoperative period [3]. In this work, we represent our experience in managing CA cases with its associated anomalies.

## MATERIALS AND METHODS

Colonic atresia cases managed at the pediatric surgery department, Ain Shams University between 2008 and 2016 were retrospectively reviewed. Gestational age, gender, age at presentation, presenting symptom, diagnostic tools, location and type of atresia, type of surgical intervention, associated anomalies, and outcome were recorded. The type of atresia was determined according to the classification of Grosfeld et al [4].

## RESULTS

Twelve patients with colonic atresia were presented and surgically managed in our department. During the same period, 244 cases of intestinal atresias were managed at our center. Colonic atresia cases accounted for 4.9 % of all gastrointestinal atresias treated in our center. The clinical data of gestational age, sex, age and type of presentation, diagnostic tool, location and type of atresia, associated anomalies are summarized in table 1. Details of surgical management, postoperative complications and outcome are reported in table 2.

**Figure:**
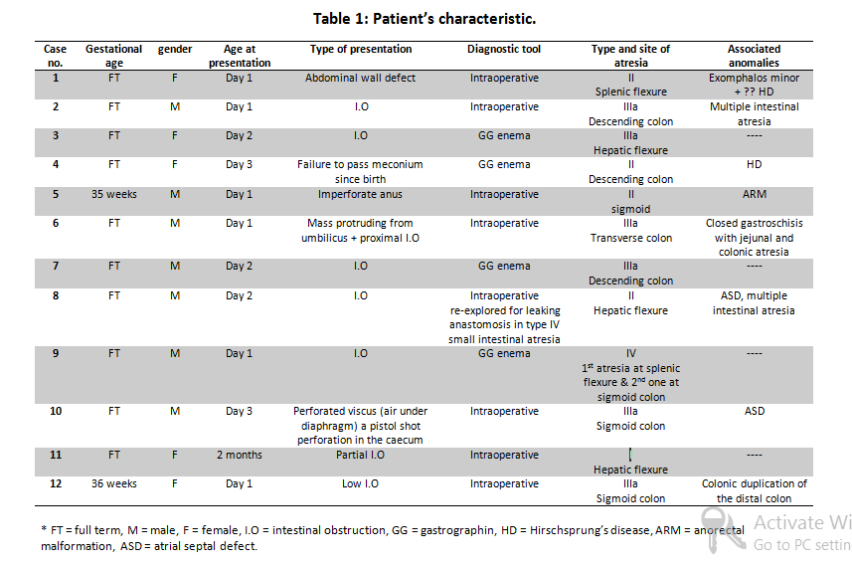
Table 1: Patient's characteristics

**Figure:**
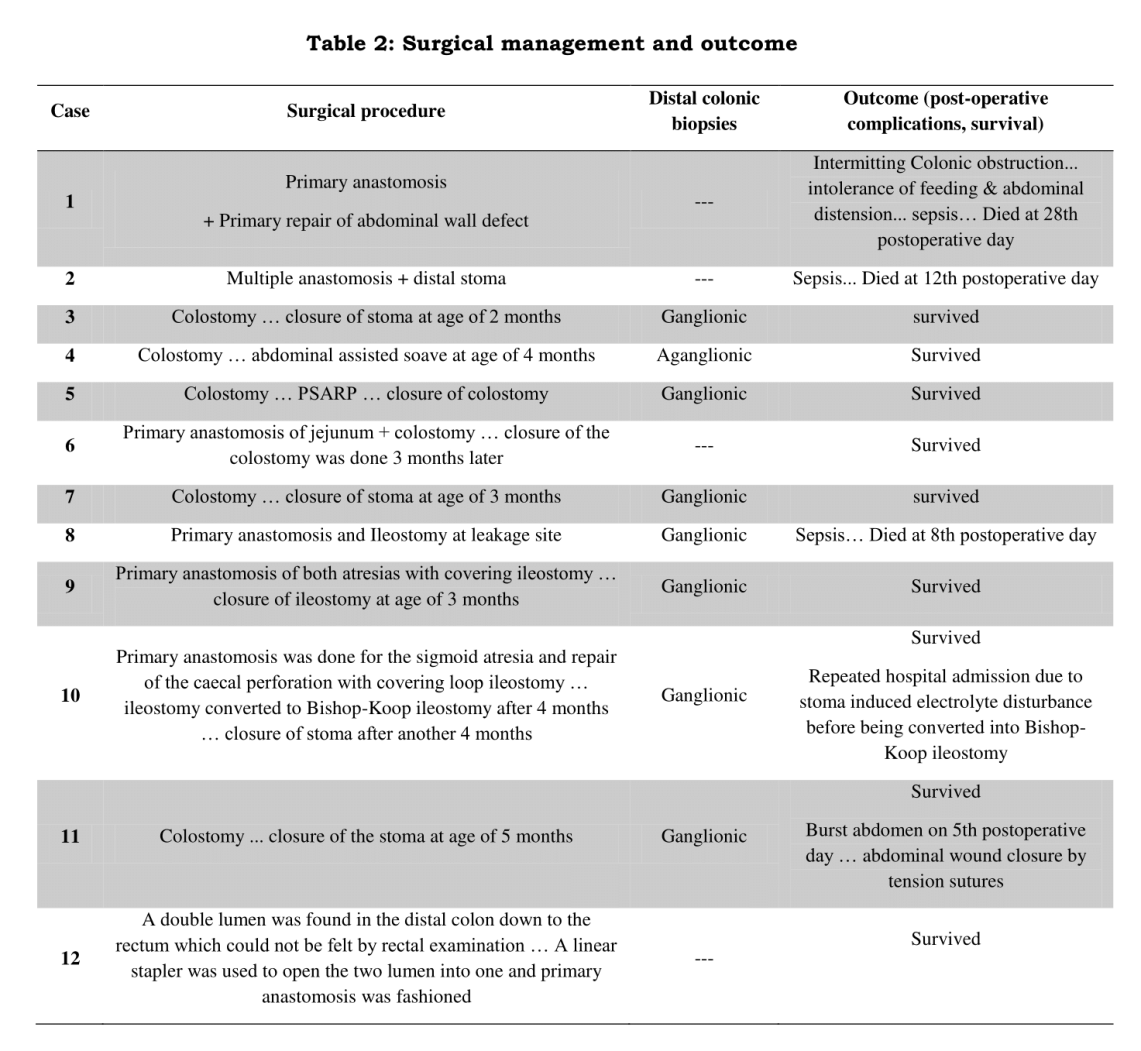
Table 2: Surgical management and outcome

The main presenting physical features were intestinal obstruction in seven cases, the associated conditions in three cases were exomphalos, closed gastroschisis, and high anomaly anorectal malformation (1 anomaly in each case), perforated viscus in one case, and partial obstruction in one case. Eight out of the twelve cases were diagnosed to have CA intraoperatively; either at exploration for low intestinal obstruction or while correcting abdominal wall defects. A diagnostic preoperative barium enema was done in four cases that revealed distal microcolon with arrest of the dye at the site of the atresia. Colonic atresias were of type IIIa in six cases, type II in four cases, type I in one case, and type IV in one case. Seven cases had associated abdominal anomalies representing 58% of cases.

Fashioning of temporary Mikulicz type stoma at the site of atresia was the initial surgical technique of choice in eight cases. Primary anastomosis was done in two cases; one of them suffered from intermittent colonic obstruction and feeding intolerance complicated by sepsis and died before exclusion of Hirschsprung's disease (HD). Primary anastomosis with covering ileostomy was done in two cases. 

Histopathologic examination of the distal colon for exclusion ofHD was done for 8 cases. It revealed aganglionosis in one female. This girl underwent abdominal assisted pull through at the age of four months. 

The overall mortality in our series was three out of 12 cases (25%). Multiple intestinal atresias were present in two mortalities; the diagnosis of CA was not made in one of them till re-exploration due to anastomotic leakage of the repaired small bowel atresia. In the third mortality, CA was associated with exomphalos who suffered from postoperative intermittent colonic obstruction and severe sepsis.

## DISCUSSION

Colonic atresia is the least common type among cases of intestinal atresias representing less than 10% of atresia cases [5]. Almost half of the reported cases were associated with other congenital anomalies. Although the pathogenesis of CA is not well known yet, there are two main different theories may explain this anomaly [6]. The classic vascular incident theory that can be caused by an intrinsic factor like thrombo-embolic event originating from the placenta and passing into the fetal circulation occluding a mesenteric blood vessels [7] or extrinsic mesenteric vascular obstruction associated with internal hernia, volvulus [8,9], or strangulation in tight gastroschisis [10]. This vascular insult occurs mostly late in gestation and leads to either the isolated phenotype of CA or the CA associated with abdominal wall defects such as gastroschisis. Colonic atresia associated with a choledochal cyst may be attributed to the intrauterine direct pressure effect of the cyst on the transverse colon mesentery [11].

The vascular theory is certainly attractive and has been reproduced in animal models. However, it could not explain the frequent association of CA with other congenital anomalies [1]. Therefore, a second theory of disturbed morphogenesis in the early embryonal period had been suggested to explain the phenotype of CA that has a variety of associated anomalies and the form of multiple intestinal atresias [6,12,13]. This theory is supported by the histological absence of Lanugo, bile pigments, and squamous epithelial cells inside the lumen of the distal loop. As the bile started to forms in the 11th week of gestation and cells can only be swallowed by the fetus after the third month of intrauterine life, so that this type of CA mostly occurred before the 12th week of life [6]. A third genetic theory is suggested by Benawra and his colleagues who reported three CA cases occurred among first-degree relatives of a family [14].

Because of the rarity of CA, proper diagnosis needs a high index of suspicion especially when facing a case of low intestinal obstruction with failure of the meconium to pass as there is always an incidence of delayed and missed diagnosis. Eight cases in our series (66.7 %) were diagnosed intraoperatively, while only four cases had established preoperative diagnosis with barium enema showing microcolon with sudden arrest of the dye at the site of atresia. In our series, we had two CA cases with delayed presentation. The first one presented at day 3 by intestinal perforation, while the second one presented at the age of two months with partial intestinal obstruction where exploration revealed a mucosal web. And we had one case with missed diagnosis of hepatic flexure type II CA that was missed in first exploration for repair of multiple small intestinal atresias. 

There is a controversy about the initial procedure for colonic atresia; to perform a primary anastomosis versus initial colostomy and staged repair which is mostly based on surgeon's experience and preference. The two main concerns when deciding to do a primary anastomosis are the discrepancy between the dilated proximal and narrow distal ends besides the possibility of associated HD in the distal loop. Karnak et al. reported their 20 years' experience for 18 CA cases where leakage had occurred in all the three cases that underwent primary anastomosis [15]. On the other side, a more recent report recommends a primary anastomosis as they document twelve neonates with CA where only two babies were managed initially with colostomy, both of whom had associated gastroschisis; while primary anastomosis was performed in ten cases with no anastomotic complications occurred and there was no mortality. However, they didn't face a case of HD in their series [16]. In our center, we preferred to fashion an initial colostomy with biopsies from distal colon to rule out HD before restoration of colonic continuity. Probably we followed this protocol because of our lost case - that we faced in our early experience - who developed undiagnosed intermittent colonic obstruction after establishing a primary anastomosis. With this strategy we could early diagnose another case of HD before restoration of intestinal continuity. 

The coexistence of CA and HD represents a diagnostic and therapeutic challenge for pediatric surgeon. About Twenty-five cases have been reported in the English-language literature [17] with reported incidence ranging from 2 to 8.4 % [2,18]. The diagnosis of coincident HD usually was made after anastomotic failure following restoration of intestinal continuity. A high index of suspicion is required to promptly diagnose HD in a child with CA. Delayed diagnosis of simultaneous colonic aganglionosis leads to a complicated postoperative course that require additional operative intervention, and prolonged hospitalization with significant increase in morbidity and mortality. To avoid anastomotic dehiscence or functional intestinal obstruction, biopsies of the distal colonic segment and/or rectum should be obtained either during the initial laparotomy or before reestablishing intestinal continuity [19].

The exact etiologies of the CA–HD association are unknown. Although this association might be coincidental, three theoretical mechanisms are described [3]. Some authors believe that CA leads to HD where the atretic segment acts as a mechanical barrier against the Cranio-caudal migration of neural crest cells into the bowel wall [20]. Thus, the aganglionic segment starts immediately distal to the atresia. However, in some infants with ileal or colonic atresia, ganglion cells were found distal to the atretic segment [3,20-23]. In a second theory, HD is believed to induce CA. A long-segment HD results in a meconium-overfilled proximal colon, which might cause an intrauterine volvulus with vascular interruption and a secondary CA. This mechanism can explain the presence of ganglion cells distal to the atretic segment [17]; however, this implies a volvulus occurring late in gestation, after the accumulation of meconium in the bowel. The third possibility is that there is a common genetic origin for both conditions [21].

Various associated congenital anomalies are reported with CA cases including both abdominal and extra-abdominal anomalies [2]. Intestinal ones are more common especially abdominal wall defects, small intestinal atresia, and malrotation [2] while other reported rare associations are choledochal cyst [11,24,25] and imperforate anus that was reported in eight cases and our case is the ninth in literature [24,26,27]. Other rare extra abdominal anomalies includes congenital heart disease, hypoplasia of the abdominal aorta, facial anomalies, and brain defects, encephalocele, ophthalmic, renal, and musculoskeletal anomalies [2,24,28,29].

The presence of associated major anomalies has a poor prognosis. The overall mortality rate in CA reported to be 25.7% in the largest review of literature [2]. Some series reported higher mortality rate up to 61 % due to either associated major anomalies or leakage from a primary anastomosis [15]. While other centers reported no mortalities in their 12 cases series although they had four cases associated with major anomalies [16]. Another important issue affecting the prognosis of these cases is the early diagnosis and management. Etensel and his colleagues observed a significant higher mortality rate with delayed diagnosis and surgical intervention for more than 72 hours from birth [2]. We had three mortalities in our series (25%); two cases were associated with multiple intestinal atresias including the case with missed diagnosis in his first exploration, and the third one who we retrospectively suspected that she had a missed diagnosis of HD.

## Conclusion

CA is associated with various intestinal and extra-intestinal anomalies which have impact on prognosis. It should be kept in mind when a pediatric surgeon faces a case of low colonic obstruction, as early diagnosis and prompt intervention is essential for better prognosis and survival. Rectal or colonic biopsy is fundamental to rule out aganglionosis before restoration of bowel continuity in CA patients. 

## Footnotes

**Source of Support:** None

**Conflict of Interest:** None
